# Impact of superselective intra-arterial and systemic chemoradiotherapy for gingival carcinoma; analysis of treatment outcomes and prognostic factors

**DOI:** 10.1186/s12885-020-07638-y

**Published:** 2020-11-26

**Authors:** Yuki Mukai, Yuichiro Hayashi, Izumi Koike, Toshiyuki Koizumi, Madoka Sugiura, Senri Oguri, Shoko Takano, Mitomu Kioi, Mizuki Sato, Kenji Mitsudo, Masaharu Hata

**Affiliations:** 1 Departments of Radiation Oncology, Yokohama City University Graduate School of Medicine, 3-9 Fukuura, Kanazawa-ku, Yokohama, 236-0004 Japan; 2 Departments of Oral and Maxillofacial Surgery, Yokohama City University Graduate School of Medicine, 3-9 Fukuura, Kanazawa-ku, Yokohama, 236-0004 Japan

**Keywords:** Gingival carcinoma, Chemoradiotherapy, Superselective intra-arterial chemoradiotherapy, External beam radiation therapy, 3-dimensional radiation therapy

## Abstract

**Background:**

We compared outcomes and toxicities between concurrent retrograde super-selective intra-arterial chemoradiotherapy (IACRT) and concurrent systemic chemoradiotherapy (SCRT) for gingival carcinoma (GC).

**Methods:**

We included 84 consecutive patients who were treated for non-metastatic GC ≥ stage III, from 2006 to 2018, in this retrospective analysis (IACRT group: *n* = 66; SCRT group: *n* = 18).

**Results:**

The median follow-up time was 24 (range: 1–124) months. The median prescribed dose was 60 (6–70.2) Gy (IACRT: 60 Gy; SCRT: 69 Gy). There were significant differences between the two groups in terms of 3-year overall survival (OS; IACRT: 78.8, 95% confidence interval [CI]: 66.0–87.6; SCRT: 50.4, 95% CI: 27.6–73.0; *P* = 0.039), progression-free survival (PFS; IACRT: 75.6, 95% CI: 62.7–85.2; SCRT: 42.0, 95% CI: 17.7–70.9; *P* = 0.028) and local control rates (LC; IACRT: 77.2, 95% CI: 64.2–86.4; SCRT: 42.0, 95% CI: 17.7–70.9; *P* = 0.015). In univariate analysis, age ≥ 65 years, decreased performance status (PS) and SCRT were significantly associated with worse outcomes (*P* < 0.05). In multivariate analysis, age ≥ 65 years, clinical stage IV, and SCRT were significantly correlated with a poor OS rate (*P* < 0.05). Patients with poorer PS had a significantly worse PFS rate. Regarding acute toxicity, 22 IACRT patients had grade 4 lymphopenia, and osteoradionecrosis was the most common late toxicity in both groups.

**Conclusions:**

This is the first report to compare outcomes from IACRT and SCRT among patients with GC. ALL therapy related toxicities were manageable. IACRT is an effective and safe treatment for GC.

## Background

Oral cancers are the 6th most common malignancy, comprising 1–3% of all malignancies [1–7]. Gingival carcinoma (GC) represents < 10% of all oral cancers in Europe and the United States, compared with 15–20% of oral cancers in Japan [1–7].

Surgical resection is the standard therapy for most oral cancers, especially early-stage disease, and is a well-established treatment for GC [2, 8, 9]. Other treatment options for oral cancers include combinations of radiation therapy (RT) including external beam RT and brachytherapy, and chemotherapy, which are considered to be organ-preserving treatments [1, 6, 8, 10]. Although brachytherapy is considered an effective organ-preserving treatment for oral cancers, particularly tongue cancer, it is often not indicated for GC because seed insertion may be challenging, and the risk of osteoradionecrosis is higher than for other oral cancers, because of the bone proximity. For unresectable cases, various chemoradiation therapies (CRT) are used, including super-selective intra-atrial, intravenous, or oral chemotherapy delivery. Intra-arterial chemoradiotherapy (IACRT) is effective for primary tumors, but might be inappropriate for distant metastases [10, 11]. However, IACRT is an effective treatment option for patients with locally advance disease without distant metastasis, patients requiring organ-preserving treatments, cases that are not suitable for brachytherapy, and for unresectable cases such as patients with advanced primary tumors or comorbidities.

Although we previously investigated the outcomes of concurrent retrograde super-selective IACRT for GC [1], few studies have summarized the use of CRT for only GC and the optimal means of administering chemotherapy in this setting is unclear. This analysis therefore compared outcomes and toxicities between patients treated with concurrent retrograde super-selective IACRT and concurrent systemic chemoradiotherapy (SCRT) for GC, based on patients from a previous study [1].

## Methods

### Patients

A total of 103 patients with GC and no distant metastasis underwent RT with curative intent at our institution between August 2006 and August 2018, of whom 84 were diagnosed as stage III or more advanced disease and were eligible for this study. The eligibility criteria were patients who received IACRT or SCRT with curative intent at our institution from 2006 to 2018, who did not receive initial surgery for the following reasons: some were considered unresectable because of primary tumor invasion; some patients with early-stage disease preferred/selected organ-preserving treatment over surgery; and some patients were unsuitable for surgery because of comorbidities, poor performance status (PS) and/or old age. Of these 84 patients, 66 received concurrent retrograde super-selective IACRT (IACRT group) and 18 received SCRT (SCRT group).

We retrospectively reviewed patients’ medical records. Clinical staging was determined by physical examination, chest X-ray, ultrasound examinations, head–pelvis computed tomography (CT), cervical magnetic resonance imaging (MRI), and positron emission tomography (PET)-CT. All patients were examined before treatment and were classified according to the International Union Against Cancer staging system and categorized according to the National Comprehensive Cancer Network (NCCN) risk classification criteria (TNM Classification of Malignant Tumors, 8th edition).

The disease characteristics of the 84 patients are summarized in Table 1. All patients were histopathologically diagnosed by biopsy of the gingiva. This study was approved by Yokohama City University Certified Institutional Review Board (B190800011, date of registration 21/10/2019), and informed consent was obtained from all patients prior to treatment. All methods were performed in accordance with the relevant guidelines and regulations.

**Table 1:** Patient characteristics

N	IACRT group	SCRT group	*P* value
The number of patients	66 (100%)	18 (100%)	
Age, years			
Median	73 (range, 46–93)	70.5 (range, 51–91)	0.26 (< 65 years)
Gender			
Male	32 (48.5%)	14 (77.8%)	0.18
Female	34 (51.5%)	4 (22.2%)	
ECOG PS			
0	50 (75.8%)	10 (55.6%)	**0.050**
1	16 (24.2%)	7 (38.8%)	
2	0	1 (5.6%)	
Histology			
Squamous cell carcinoma	63 (95.5%)	17 (94.4%)	NA
Verrucous carcinoma	1 (1.5%)	1 (5.6%)	
Poorly differentiated carcinoma	2 (3%)	0	
Clinical stage (TNM Classification of Malignant Tumors, 8th edition)			
Stage III	14 (21.2%)	3 (16.7%)	0.68
Stage IVA	49 (74.3%)	13 (72.2%)	
Stage IVB	3 (4.5%)	2 (11.1%)	
T category			0.27
T2	7 (10.6%)	2 (11.1%)	
T3	17 (25.8%)	2 (11.1%)	
T4a	37 (56.0%)	11 (61.1%)	
T4b	5 (7.6%)	3 (16.7%)	
N category			0.22
N0	25 (37.9%)	4 (22.2%)	
N1	12 (18.2%)	4 (22.2%)	
N2a	0	1 (5.6%)	
N2b	22 (33.3%)	3 (16.7%)	
N2c	5 (7.6%)	6 (33.3%)	
N3a	2 (3%)	0	
Location			
Lt	35 (53.0%)	12 (66.7%)	0.34
Rt	25 (37.9%)	5 (27.7%)	
Middle	6 (9.1%)	1 (5.6%)	
Upper/maxillary	41 (62.1%)	7 (38.8%)	0.19
Lower/mandibular	25 (37.9%)	11 (61.2%)	
Tumor diameter (maximum, mm)	40 (range, 12–60)	45 (range, 20–90)	0.31

Comparison of clinical variables between the two groups was performed using Mann-Whitney’s U-test

*EOCG PS* Eastern Cooperative Oncology Group performance status

### Treatment

According to our previously published study [1], patients received external irradiation at a planned total dose of 60–70 Gy in 30–35 fractions using three-dimensional RT. The fraction size was 2 Gy delivered daily, 5 days per week, using 6 MV X-rays and a shrinking field technique. Gross tumor volume (GTV) was defined as the primary tumor and the metastatic lymph nodes, the clinical target volume (CTV) was defined as GTV plus 5 mm margins, and the planning target volume was defined as CTV plus 5–10 mm margins.

**Fig. 1:**
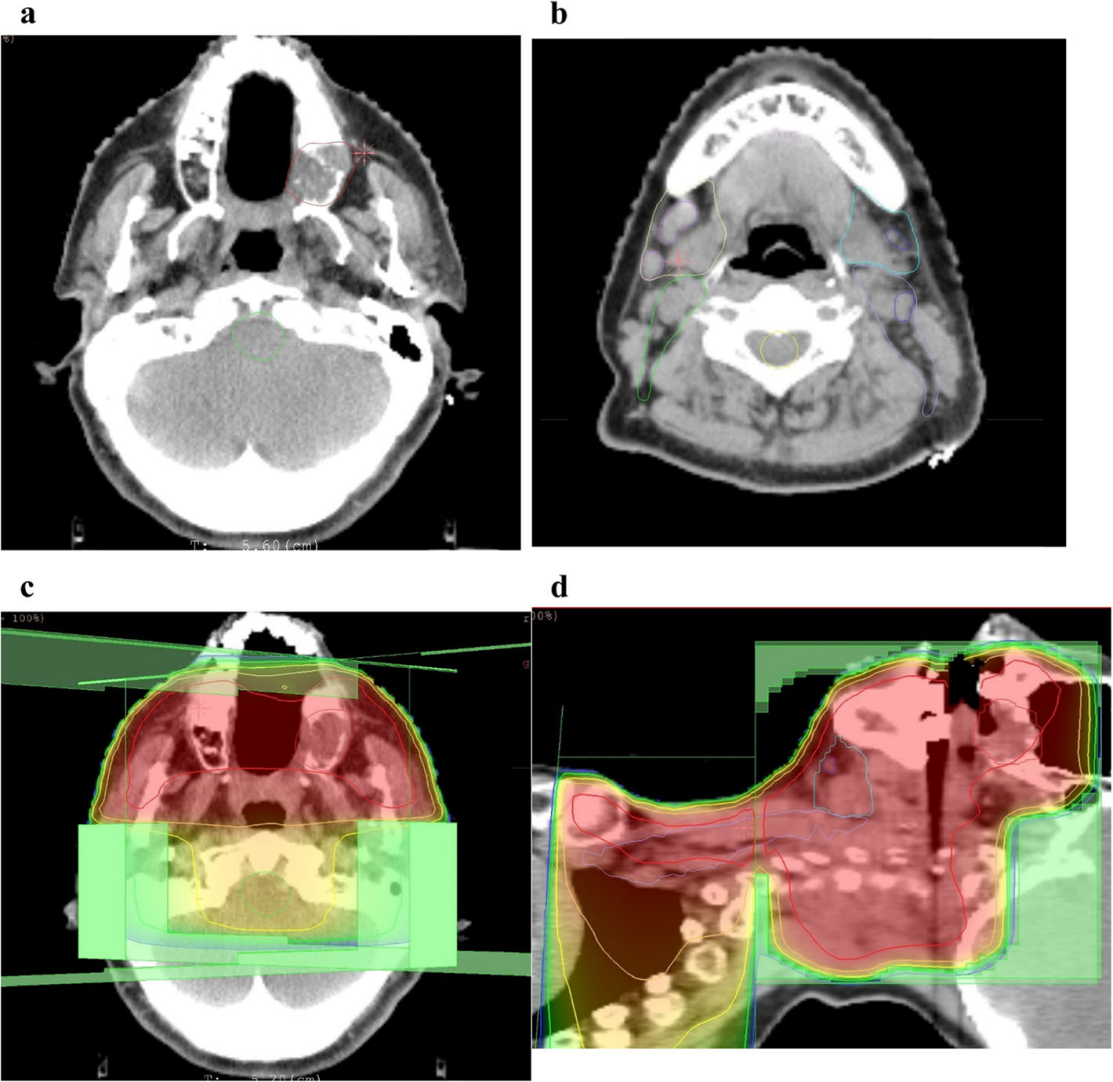
Contouring of (**a**) primary tumor and (**b**) lymph node areas. (**c, d**) Dose distribution. Red lines indicate 100% of prescription dose, orange 85%, yellow 70%, green 55%, and blue 40%

In patients with no cervical lymph node metastasis and T2–4 primary tumors, the radiation field was set up to include the primary tumors and prophylactically the ipsilateral cervical lymph node area (levels I–III). Patients with cervical lymph node metastasis underwent irradiation of the primary tumors and the ipsilateral (levels I–IV for N1) or bilateral (levels I–V for ≥N2) cervical lymph node area, including metastatic lymph nodes. After a total dose of 40 Gy had been delivered to the initial field, an additional 20–30 Gy was delivered to the GTV within the shrunken field. Prophylactic cervical irradiation was delivered in 40 Gy doses. The total dose to the spinal cord was restricted to 45 Gy in all patients. An example of contouring and dose distribution is shown in Fig. 1.

### Chemotherapy

In the IACRT group, catheters were placed via the superficial temporal artery and occipital artery and CT angiography with indigo-carmine injection was initially performed to detect tumor-feeding arteries and details of tumor invasion. Primary tumors are often fed by maxillary artery and facial artery. In cases where the mandibular gingival tumor had spread to the lingual side or the mouth floor, the lingual artery was catheterized. Chemotherapy was started at the same time as RT. The most frequently chemotherapy regimen was that cisplatin (5 mg/m^2^/day) and docetaxel (DOC; 10 mg/m^2^/week) administered by infusion over a period of 1 h, 5 days per week as a single course, for 6 weeks. Patients who received IACRT had no history of cerebral infarction; liver, kidney or heart dysfunction, or severe diabetes mellitus. In the SCRT group, 18 patients had chemotherapy with RT consisting of tegafur/gimeracil/oteracil potassium (TS-1, 80–120 mg/day, *n* = 9), cetuximab (250–400 mg/m^2^, *n* = 6), tegafur–uracil (UFT; 300 mg/m^2^, *n* = 2), or DOC (60 mg/m^2^) + fluorouracil (100 mg; *n* = 1). The period of initial chemotherapy, in the SCRT group was the same as that of RT.

### Evaluation criteria and statistical analysis

Responses were evaluated using clinical examination and CT, MRI and PET-CT studies at approximately 4–6 weeks after completing treatment. Tumor responses were assessed using Response Evaluation Criteria in Solid Tumors (ver. 1.1) [12]. If residual metastatic lymph nodes were suspected after treatment, radical neck dissection was planned. Toxicities associated with treatments were evaluated using the Common Terminology Criteria for Adverse Events, v4.03 [13]. Acute toxicities were defined as therapy-related adverse events that occurred within 3 months after starting treatment, and late toxicities as those occurring after 3 months.

We compared clinical variables between the two groups using the Mann–Whitney U-test. Overall survival (OS), local control (LC), and progression-free survival (PFS) rates from the beginning of treatment were calculated with Kaplan–Meier curves. Differences between curves were tested by the log-rank test. Analyses of prognostic factors was carried out using univariate and multivariate Cox proportional-hazards regression models, with the Statistical Package for the Social Sciences (SPSS for Windows, version 23.0; IBM, Armonk, NY, USA). *P* < 0.05 was considered significant.

## Results

### Treatment

The median follow-up time was 24 (range, 1–124) months in all patients. Treatment characteristics of the study group are summarized in Table 2.The reasons for patients not receiving IACRT included the following: difficulty in using contrast medium because of renal or liver dysfunction or allergy (*n* = 4); difficulty in placing/inserting catheters because of dementia or anticoagulant use (*n* = 7); and advantage or preferability of systemic chemotherapy because of comorbidities (e.g., history of another carcinoma or tuberculosis, *n* = 6). One patient switched to systemic chemotherapy because she had a stroke after catheter placement. The completion rates for IACRT and SCRT were 90.9 and 88.9%, respectively. Six IACRT patients (9.1%) and two SCRT patients (11.1%) discontinued RT because of complications/coexisting disease (including infection, bleeding from gastrostomy, or delirium) or at the patient’s request.

**Table 2:** Treatment related characteristics

N	IACRT group	SCRT group	*P* value
Overall treatment time for radiation therapy (month)	46.5 (range,8–73)	52 (range,2–94)	0.090
Total radiation dose (Gy)	60 (range, 14–70.2)	69 (range, 6–70.2)	**0.013**
Radiation dose for prophylactic cervical LN node area (Gy)	40 (range, 20–41.4)	40 (range, 6–40)	0.65
RT field Ipsilateral cervical LN area Bilatera cervical LN area Only primary tumor	Ipsilateral: 17 (25.8%) Bilateral: 45 (68.2%) Primary tumor: 4 (6.0%)	Ipsilateral: 3 (16.6%) Bilateral: 14 (77.8%) Primary tumor: 1 (5.6%)	0.59
Chemotherapy	CCDP+DOC: 61 (92.5%) CDDP+5FU: 1 (1.5%) CDDP+DOC+cetuximab:2 (3.0%) CDDP only: 1 (1.5%) CDDP+ cetuximab: 1 (1.5%)	TS-1: 9 (50%) UFT:2 (11.1%) DOC+5FU: 1 (5.6%) Cetuximab: 6 (33.3%)	NA
Gastrostomy	36 (54.5%) Gastric tub: 13 (19.7%) Ingestion/oral intake17 (25.8%)	4 (22.2%) Gastric tub: 3 (16.7%) Ingestion/oral intake:11 (61.1%)	0.005

Comparison of clinical variables between the two groups was performed using Mann-Whitney’s U-test

*RT* radiation therapy, *LN* lymph node, *CCDP* cisplatin, *DOC* docetaxel, *NA* not applicable

### Tumor control

Following initial therapy, 14 patients in the IACRT group and 10 in the SCRT group had residual disease within the RT field. In the IACRT group, 10 patients with residual primary tumors received salvage therapy, which resulted in control in three. Salvage therapy included surgery for primary tumors or neck dissection (*n* = 3), additional RT (e.g., stereotactic body radiation therapy, *n* = 4) and additional chemotherapy (*n* = 3). In the SCRT group, four patients received salvage therapy, but none achieved disease control.

**Fig. 2:**
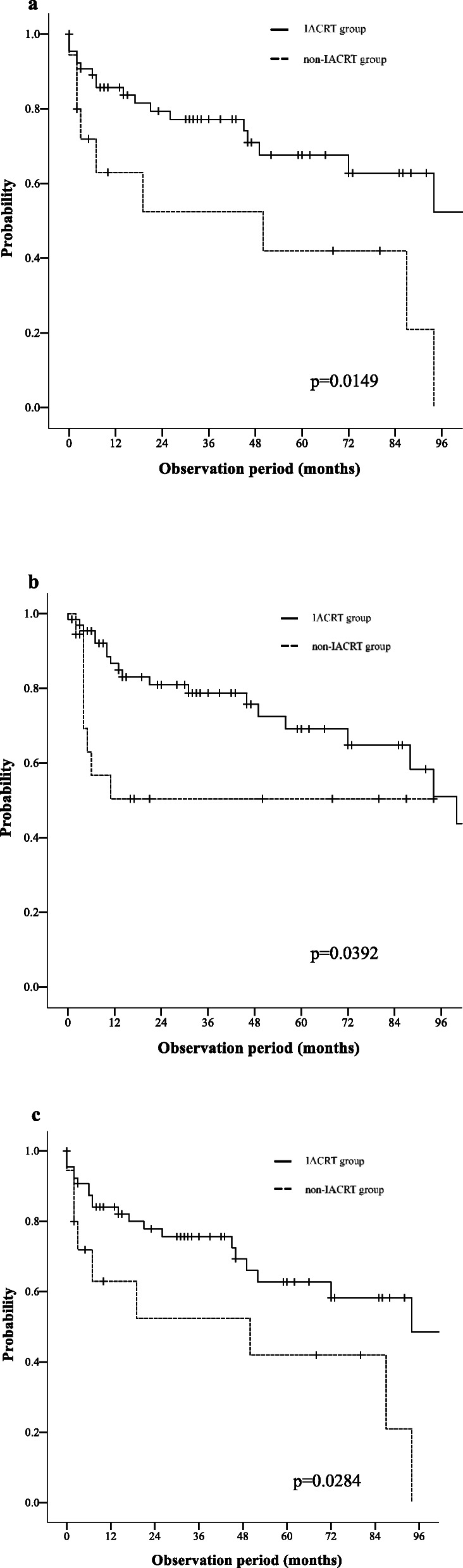
**a**. Local control. **b**. Overall survival. **c**. Progression-free survival

Among IACRT patients whose primary tumors were considered to be controlled, four patients underwent neck lymph node dissections, and three of these four patients had no evidence of malignancy in their neck lymph nodes. The 3-year LC rate differed significantly between the IACRT group (77.2%; 95% confidence interval [CI]: 64.3–86.4) and the SCRT group (42.0%; 95% CI: 17.7–70.9) (*P* = 0.015) (Fig. 2a).

Regarding recurrence, 10 patients in the IACRT group and one in the SCRT group developed recurrence within the RT field, and eight patients in the IACRT group and four in the SCRT group had distant metastases. Seven IACRT patients (10.6%) developed primary tumor recurrences, compared with no SCRT patients. Five IACRT patients (7.6%) developed cervical lymph node recurrences compared with only one SCRT patient (5.6%). Eight IACRT patients (12.1%) experienced distant metastases (lung metastases with or without other sites: *n* = 6, pleural dissemination or subcutaneous metastasis: *n* = 2) compared with four SCRT patients (22.2%; lung metastases: *n* = 3, liver metastases: *n* = 1). The lung metastasis rates in the IACRT and SCRT groups were 9.1 and 16.7%, respectively. Four IACRT patients who received salvage therapy after recurrence were alive at the last follow-up.

### Survival

The two groups differed significantly in terms of 3-year OS (IACRT: 78.8, 95% CI: 66.0–87.6; SCRT: 50.4, 95% CI: 27.6–73.0; *P* = 0.039) and PFS rates (IACRT: 75.7, 95% CI: 62.7–85.2; SCRT: 42.0, 95% CI: 17.7–70.9; *P* = 0.028) (Fig. 2b and c). Among the 66 IACRT patients, 47 patients were alive, and 43 were considered achieved CR (median survival: 46 months, range: 7–124 months) at May 31, 2019. Of the 19 IACRT patients who died within 1–56 months after treatment, 16 died of cancer and three died of non-cancer-related causes. In the SCRT group, only four had CR (median follow-up time: 84.5 months, range: 50–94 months). Nine patients died (6 of cancer and 3 of non-cancer-related causes) and five patients changed hospital to receive palliative care. Neither group had any therapy-related mortality.

### Analysis of prognostic factors

Univariate and multivariate analyses of factors associated with OS, PFS and LC rates are summarized in Tables 3 and 4. In univariate analysis, 65 years or older of age, poorer PS, and SCRT treatment were significantly associated with worse OS rate (*P* = 0.003, *P* = 0.024, *P* = 0.047, respectively). Among patients aged 65 years or older, clinical stage IV, > T3, > N2b, and SCRT treatment were significantly associated with worse PFS and worse LC rates (*P* = 0.033, *P* = 0.029, *P* = 0.041, *P* = 0.004, *P* = 0.048, respectively). A prescribed dose > 60 Gy was significantly associated with a worse PFS rate (*P* = 0.038).

**Table 3:** Univariate analysis for prognostic factors

Variables		OS			PFS			LC		
		HR	95% CI	*P* value	HR	95% CI	*P* value	HR	95% CI	*P* value
Age, years	< 65	1/reference			1			1		
	≥ 65	4.655	1.389–15.59	**0.003**	2.618	0.992–6.906	**0.033**	3.1035	1.067–9.028	**0.019**
Gender	Female	1			1			1		
	Male	2.021	0.906–4.505	*0.076*	1.312	0.630–2.733	0.466	1.324	0.618–2.833	0.468
ECOG PS	0	1			1			1		
	> 1	2.555	1.168–5.589	**0.024**	2.013	0.925–4.382	0.091	1.893	0.8399–4.265	0.139
Clinical stage	≤ stage 3	1			1			1		
	> stage 4	2.583	0.874–7.632	*0.058*	2.905	0.994–8.490	**0.029**	3.715	1.099–12.56	**0.014**
T category	≤ T3	1			1			1		
	> T3	1.204	0.548–2.649	0.642	2.316	0.984–5.453	**0.041**	2.567	1.0298–6.399	**0.029**
N category	≤ N2b	1			1			1		
	> N2b	2.794	1.077–7.252	*0.052*	4.117	1.722–9.845	**0.004**	4.430	1.831–10.72	**0.003**
Location	Lower	1			1			1		
	Upper	0.909	0.423–1.950	0.806	0.796	0.382–1.658	0.542	0.7923	0.370–1.695	0.548
Chemotherapy	IACRT	1			1			1		
	SCRT	2.34	1.012–5.41	**0.047**	2.334	1.058–5.149	**0.048**	2.586	1.156–5.786	**0.030**
RT field	primary tumor	1			1			1		
	cervical LN area	0.727	0.171–3.098	0.680	1.492	0.348–6.390	0.6099	1.591	0.369–6.858	0.558
Prescribed total dose	≤60 Gy	1			1			1		
	> 60 Gy	1.003	0.438–2.299	0.993	2.225	1.066–4.65	**0.0378**	2.177	1.015–4.672	*0.0515*

**Table 4:** Multivariate analysis for prognostic factors

Variables		OS			PFS			LC		
		HR	95% CI	*P* value	HR	95% CI	*P* value	HR	95% CI	*P* value
Age, years	< 65	1			1			1		
	≥ 65	3.645	1.041–12.77	**0.022**	1.835	0.671–5.015	0.212	2.210	0.732–6.675	0.130
ECOG PS	0	1			1			1		
	> 1	1.956	0.807–4.738	0.144	2.865	1.125–7.298	**0.032**	2.521	0.935–6.801	*0.074*
Clinical stage	≤ stage 3	1			1			1		
	> stage 4	6.735	1.581–28.70	**0.009**	1.866	0.368–.465	0.459	2.609	0.462–14.73	0.284
T category	≤ T3	1			1			1		
	> T3	1.921	0.677–5.469	0.236	1.618	0.452–.785	0.437	1.513	0.417–5.480	0.512
N category	≤ N2b	1			1			1		
	> N2b	1.425	0.513–3.957	0.506	2.468	0.966–6.301	*0.069*	2.415	0.936–6.229	*0.078*
Chemotherapy	IACRT	1			1			1		
	SCRT	3.161	1.055–9.468	**0.041**	1.389	0.524–3.686	0.511	1.630	0.595–4.463	0.344
Prescribed total dose	≤60 Gy	1			1			1		
	> 60 Gy	1.992	0.718–5.533	0.174	1.715	0.674–4.359	0.262	1.438	0.543–3.808	0.468

*OS* overall survival, *LC* local control, *PFS* progression-free survival, *EOCG PS* Eastern Cooperative Oncology Group performance status, *RT* radiation therapy, *IACRT* Intra-arterial chemoradiation, *SCRT* systemic chemoradiation therapy

In multivariate analysis, 65 years or older of age, clinical stage IV, and SCRT group were significantly correlated with poor OS rates (*P* = 0.022, *P* = 0.009, *P* = 0.041, respectively). Patients with poorer PS had significantly worse PFS rate (*P* = 0.032).

### Toxicities

Therapy-related acute toxicities are shown in Table 5. The two groups differed significantly in the incidences of grade 3 leukopenia (*P* = 0.012), radiation dermatitis (*P* = 0.034), and dysphagia (*P* = 0.008). Interestingly, 22 IACRT patients had grade 4 lymphopenia, but recovered immediately after treatment. In both groups, osteoradionecrosis (mandibular: *n* = 13; maxillary: *n* = 2) was the most common late toxicity, affecting nine IACRT patients (13.6%; median: 32 months; range: 17–107 months) and six SCRT patients (33.3%; median 43 months; range: 3–16 months) (*P* = 0.081). One IACRT patient developed a pharyngeal fistula and required surgery (grade 4). No SCRT patient developed any other late severe (≥ grade 3) toxicity associated with treatment.

**Table 5:** Acute toxicity

Acute Toxicities	IACRT group				SCRT group				*P* value
	≤ Grade 1	Grade 2	Grade 3	≥ Grade 4	≤ Grade 1	Grade 2	Grade 3	≥ Grade 4	≥ Grade 3
***Hematologic toxicity***									
Anemia	21 (31.8%)	35 (53.1%)	10 (15.1%)	0	6 (33.3%)	11 (61.1%)	1 (5.6%)	0	0.24
leukopenia	23 (34.8%)	19 (28.8%)	19 (28.8%)	5 (7.6%)	14 (77.7%)	3 (16.7%)	1 (5.6%)	0	**0.012**
Neutropenia	37 (56.1%)	15 (22.7%)	13 (19.7%)	1 (1.5%)	17 (94.4%)	0	1 (5.6%)	0	0.13
lymphopenia	6 (9.1%)	12 (18.2%)	26 (39.4%)	22 (33.3%)	7 (38.8%)	1 (5.6%)	10 (55.6%)	0	0.21
thrombopenia	61 (92.5%)	2 (3.0%)	3 (4.5%)	0	18 (100%)	0	0	0	0.37
***General conditions***									
Radiation dermatitis	25 (37.9%)	27 (40.9%)	12 (18.2%)	2 (3.0%)	8 (%)	10 (%)	0	0	**0.034**
Oral mucositis	2 (3.0%)	20 (30.3%)	43 (65.2%)	1 (1.5%)	3 (16.7%)	7 (38.8%)	7 (38.8%)	1 (5.6%)	0.088
Xerostomia	43 (65.2%)	20 (30.3%)	2 (3.0%)	0	10 (55.6%)	8 (44.4%)	0	0	0.46
Dysphagia	0	17 (25.8%)	49 (74.2%)	0	0	11 (61.1%)	7 (38.8%)	0	**0.008**

*IACRT* Intra-arterial chemoradiation, *SCRT* systemic chemoradiation therapy

## Discussion

Surgery remains the standard curative treatment for oral cancer [1]. However, gingival tumors are often adjacent to the maxillary or mandibular bone, and mandibulectomy or maxillectomy may be required to secure adequate margins [14–17]. Patients with GC may thus suffer diminished quality of life after surgery, caused by oral dysfunction and cosmetic impairment. To the best of knowledge, this is the first study to compare intra-arterial chemoradiotherapy, and systemic chemoradiotherapy for GC. Although RT with intravenous or intra-arterial chemotherapy has previously been reported as an organ-preserving treatment for advanced oral carcinoma [1, 3], there are currently no prospective studies or established evidence for the use of these treatments for GC.

Lubek et al. reported that a cohort with GC (of whom half had stage I–II disease) treated with surgery alone had a 5-year OS rate of 38% [4], while patients with lower/mandibular GC treated by surgery alone had 5-year cause-specific and disease-specific survival rates of 73 and 80.6%, respectively [3, 18, 19]. Among patients who received postoperative RT for GC, including stage I–IV disease, the reported 5-year OS and disease-free survival rates were 36.5–95% and 21.1–68%, respectively [6, 20]. Lank et al. compared definitive RT and postoperative RT using intensity-modulated radiation therapy for stage III–IV GC and found a 5-year OS rate of 36.6%; however, only 53.9% patients in their study received concurrent chemotherapy or immunotherapy [20].

In the current study, the 3-year OS, PFS and LC rates in the IARCT and SCRT groups were 78.8 and 50.4%, 75.6 and 42.0%, and 77.2 and 42.0%, respectively. Comparisons with previous studies may be difficult because of their differing populations and stages. However, the overall outcome of the present study was comparable to or better than previous reports of surgery and postoperative RT [3, 4, 6, 18–20]. Other reports on IACRT-treated oral cancers reported distant metastasis rates of 10–23% [10, 11], whereas the rate in the present study was lower in the IACRT group (12.1%) than in the SCRT group (22.2%), and half of the patients who developed distant metastasis did not have controlled primary tumors. Furthermore, three of four (75%) IACRT patients were pathologically confirmed to have no evidence of malignancy in their neck lymph nodes after lymph node dissection. IACRT may be disadvantageous with respect to distant or locoregional (lymph node) metastasis because this method distributes chemotherapy drugs to relatively limited areas. Our IACRT and SCRT groups showed significantly different in OS (*P* = 0.039), PFS (*P* = 0.028), and LC rates (*P* = 0.015) at 3 years. Moreover, in multivariate analysis, IACRT was significantly correlated with better OS rate (*P* = 0.041). These results suggest that intensive local therapy for primary tumors leads to better control of metastasis. The present study found that RT doses > 60 Gy were significantly associated with worse PFS rates in univariate analysis. The median prescribed dose was higher in the SCRT group (69 Gy) than in the IACRT group (60 Gy) (*P* = 0.013). The 2019 NCCN guidelines recommended 66–70 Gy as the definitive RT dose for cancer of the oral cavity; however, this is not a recommendation for IACRT. In our study, an additional dose > 60 Gy was prescribed in cases with macroscopic residual tumor in the latter half of the RT; however, dose-escalation > 60 Gy may be not effective in IACRT in these cases. This may account for the apparent discrepancy with the international treatment guidelines.

Among therapy-related acute toxicities, ≥ grade 3 leukopenia, radiation dermatitis, and dysphagia rates were higher in the IACRT group than in the SCRT group. However, they were all transient and the completion rate was almost 90% for both treatments. Watanabe et al. compared osteoradionecrosis rates between intravenous CRT and IACRT in various oral cancers, including a few GCs, and reported a higher, but not significant, incidence in the IACRT group (CRT 10%; IACRT 17%) [21]. They suggested that prophylactic dental care is effective against radiation-induced toxicity. We did not expect the osteoradionecrosis rate to be lower in the IACRT group (13.6%) compared with the SCRT group (33.3%) (*P* = 0.081), because we previously found the opposite situation [1]. We suppose that oral hygiene for the IACRT patients in our study may have been carefully managed, which may have led to the lower osteoradionecrosis rate in the IACRT group.

One patient developed a stroke after catheter placement. Her only complication at that time was hypertension, and the direct cause of her stroke was unclear. Eight patients terminated RT, mostly because of infection (catheter or enteritis) (37.5%). Given that the IACRT group had a higher rate of leukopenia than the SCRT group, the risk of infection may also have been higher. Marta et al. reported that the GC patients with diabetes mellitus had a higher recurrence rate after postoperative RT [22]. Taken together, we suggest careful management of oral hygiene and infection monitoring during IACRT, and careful follow-up for patients with diabetes mellitus after treatment.

This study had several limitations, including its retrospective design, the much greater size of the IACRT group compared with the SCRT group, and the relatively short overall follow-up time. The reasons why patients did not receive IACRT are given in the Results section, but the selection of patients to receive IACRT or SCRT represents a major bias of this study. Moreover, patients with poorer PS had significantly different PFS rates in multivariate analysis (*P* = 0.032), and PS differed significantly between the two groups (*P* = 0.050); this should therefore be considered when interpreting our results.

## Conclusion

IACRT led to significantly better 3-year outcomes than SCRT in patients with GC and was an independent predictor of better OS rates in multivariate analysis. IACRT is thus an effective and safe treatment for GC; however, further studies are needed to determine its long-term efficacy and late toxicities. Nevertheless, IACRT is an effective organ-preserving treatment choice for patients with GC.

## Data Availability

The datasets used and/or analysed during the current study are available from the corresponding author on reasonable request.

## Abbreviations

GC: Gingival carcinoma
RT: Radiation therapy
CRT: Chemoradiation therapies
IACRT: Intra-arterial chemoradiation
SCRT: Systemic chemoradiation therapy
CT: Computed tomography
MRI: Magnetic resonance imaging
NCCN: National Comprehensive Cancer Network
GTV: Gross tumor volume
CTV: Clinical target volume
PTV: Planning target volume
RECIST: Response Evaluation Criteria in Solid Tumors
CTCAE: Common Terminology Criteria for Adverse Events
OS: Overall survival
LC: Local control
PFS: Progression-free survival
SPSS: Statistical Package for the Social Sciences
CR: Complete response
QOL: Quality of life
DFS: Disease-free survival
IMRT: Intensity-modulated radiation therapy
